# Gynecologic and obstetric complications in women with congenital fibrinogen disorders: insights from the Prospective Rare Bleeding Disorders Database

**DOI:** 10.1016/j.rpth.2025.102960

**Published:** 2025-06-27

**Authors:** Samin Mohsenian, Roberta Palla, Marzia Menegatti, Andrea Cairo, Simona Maria Siboni, Marguerite Neerman-Arbez, Mehran Karimi, Helen Pargantou, Rosanna Asselta, Danijela Mikovic, Marko Saracevic, Britta Laros-van Gorkom, Laura Jacobs, Amy Shapiro, Adrianna Williamson, Michael Makris, Alessandro Casini, Flora Peyvandi

**Affiliations:** 1 Università degli Studi di Milano, Department of Pathophysiology and Transplantation, Milan, Italy; 2 Fondazione IRCCS Ca’ Granda Ospedale Maggiore Policlinico, Angelo Bianchi Bonomi Hemophilia and Thrombosis Center and Fondazione Luigi Villa, Milan, Italy; 3 Department of Genetic Medicine and Development, Faculty of Medicine, University of Geneva, Geneva, Switzerland; 4 Pediatric Hematology-Oncology Department, American Hospital Dubai, Dubai, United Arab Emirates; 5 Hematology Research Center, Shiraz University of Medical Sciences, Shiraz, Iran; 6 Haemophilia Centre-Haemostasis and Thrombosis Unit, Aghia Sophia Children’s Hospital, Athens, Greece; 7 Department of Biomedical Sciences, Pieve Emanuele, Humanitas University, Milan, Italy; 8 IRCCS Humanitas Research Hospital, Rozzano, Milan, Italy; 9 Hemostasis Department, Blood Transfusion Institute of Serbia, Belgrade, Serbia; 10 Department of Hematology, Radboud University Medical Center, Nijmegen, the Netherlands; 11 Indiana Hemophilia and Thrombosis Center, Indianapolis, Indiana, USA; 12 Indiana Hemophilia and Thrombosis Center, Innovative Hemophilia, Indianapolis, Indiana, USA; 13 Sheffield Haemophilia and Thrombosis Centre, Royal Hallamshire Hospital, Sheffield, UK; 14 Division of Angiology and Hemostasis, Faculty of Medicine, Geneva University Hospitals, Geneva, Switzerland

**Keywords:** bleeding, *FGA*, *FGB*, FGG, fibrinogen, genetic, gynecological, hypofibrinogenemia, miscarriage, obstetrical, postpartum hemorrhage

## Abstract

**Background:**

Women and girls with congenital fibrinogen deficiencies (CFDs) face higher hemorrhagic risks during their reproductive years, yet data on gynecologic and obstetric complications remain limited.

**Objectives:**

We aimed to rate the prevalence of heavy menstrual bleeding and obstetric complications in women with CFDs and compare our findings with previous reports.

**Methods:**

This study analyzed data from the Prospective Rare Bleeding Disorders Database registry, including available fibrinogen activity and antigen levels, as well as clinical phenotype and genotype (2013-2020).

**Results:**

A total of 59 women (8 afibrinogenemic, 15 hypofibrinogenemic, and 36 dysfibrinogenemic cases) were investigated, of which 32 patients had 70 pregnancies. The prevalence of heavy menstrual bleeding was comparable between hypofibrinogenemic (27%) and dysfibrinogenemic (36%) cases, with a higher frequency in afibrinogenemic (75%) cases. The rates of postpartum hemorrhage at 36% and miscarriage at 23% were notably higher than those observed in the general population (1%-10% and 10%-20%, respectively). These complications were similarly distributed among patients with dysfibrinogenemia (35% and 37%) and hypofibrinogenemia (36% and 31%). There were only 2 (4%) bleeds during pregnancy, both occurring in dysfibrinogenemic cases. Miscarriage was also observed in 50% of the asymptomatic dysfibrinogenemic patients. No significant difference in miscarriage and postpartum hemorrhage rates was found between dysfibrinogenemic individuals with and without hotspot variants (*P* = .94).

**Conclusion:**

The high rate of obstetric complications in women with CFDs highlights the need for early diagnosis and the potential need for prophylaxis, as pregnancy may also pose risks in asymptomatic cases. Hotspot variants do not appear to increase the risk of obstetric complications.

## Essentials

• Women and girls with CFD may face more adverse outcomes.
• This study used data from the Prospective Rare Bleeding Disorders Database.
• In qualitative CFDs, live birth, PPH, and miscarriage rates were 64%, 23%, and 36%.
• Early diagnosis and prophylaxis before pregnancy may help prevent obstetric complications.

## 1 Background

Congenital fibrinogen deficiencies (CFDs) are inherited bleeding disorders resulting from variants in the fibrinogen genes *FGA*, *FGB*, and *FGG* [1]. The disorder is categorized into quantitative (afibrinogenemia and hypofibrinogenemia) and qualitative (dysfibrinogenemia and hypodysfibrinogenemia) defects based on plasma fibrinogen levels [2]. Afibrinogenemia is characterized by the near-complete absence of fibrinogen in plasma, whereas hypofibrinogenemia involves a proportional reduction in both fibrinogen coagulant activity and antigen (fibrinogen activity/antigen >0.7). Dysfibrinogenemia, in contrast, is defined by reduced fibrinogen activity despite normal antigen levels, while hypodysfibrinogenemia is characterized by disproportionate reductions in both fibrinogen activity and antigen (fibrinogen activity/antigen <0.7) [2,3]. Depending on factor plasma levels or genetic makeup, patients with CFDs exhibit a wide range of clinical symptoms, ranging from severe bleeding to being entirely asymptomatic or even experiencing thrombotic events [4,5].

Women and girls with CFDs may experience more adverse outcomes given their physiological exposure to hemorrhagic risk during the reproductive age. Heavy menstrual bleeding (HMB) is often the most common symptom, and in some cases, the sole clinical manifestation of CFDs [6,7]. Pregnancy poses additional challenges for affected women because fibrinogen is a critical protein required for maintaining the placenta, supporting early trophoblast proliferation and spreading, thereby contributing to successful pregnancies [8,9]. A previous study indicated that women with afibrinogenemia and dysfibrinogenemia face a heightened risk of placental pathologies, such as abruption [10]. In contrast, in the Fibrinogest study, placental abruption (especially early in pregnancy) was more common in women with hypofibrinogenemia [11]. Additionally, the risk of miscarriage and bleeding during pregnancy is well documented in those with severe CFDs [12]. Due to the high rate of early miscarriage in women with afibrinogenemia and severe hypofibrinogenemia, a fibrinogen concentrate should be administered soon after pregnancy confirmation and must be continued during pregnancy to maintain a fibrinogen plasma level at ≥ 100 mg/dL [13,14]. Women with severe or moderate hypofibrinogenemia or dysfibrinogenemia, particularly those carrying prothrombotic variants, encounter a significantly increased risk of miscarriage due to low or dysfunctional fibrinogen. The prevalence of miscarriage in this population is reported to range from 20% to 50% [5,15,16], which is notably higher than the worldwide risk of miscarriage, which is approximately 15.3% [17]. Women and girls with CFDs are at increased risk of bleeding due to their physiological exposure to hemorrhagic challenges during menstruation, pregnancy, childbirth and in the postpartum period. Despite these potential complications, data on gynecologic and obstetric outcomes in individuals with CFDs remain scarce. Understanding the impact of these disorders on pregnancy, miscarriage risk, and postpartum hemorrhage (PPH) is crucial for improving clinical management and developing preventive strategies.

Hence, in this study, we aimed to assess the rate of gynecologic and obstetric complications in women and girls with CFDs using the Prospective Rare Bleeding Disorders Database (PRO-RBDD). This database compiled demographic and clinical data from birth to enrollment, providing insights into the medical history of affected individuals [7,18].

## 2 Material and Methods

This study analyzed baseline data recorded in the PRO-RBDD [7], encompassing 59 female participants of reproductive age (≥12 years old) with available fibrinogen activity and antigen levels, as well as clinical manifestation and genotype information, collected between 2013 and 2020. We also included women above the typical fertile age (eg, >50 years), as they may have experienced relevant gynecologic complications prior to the age at inclusion. The historical diagnosis of fibrinogen deficiency was based on coagulant activity levels below the normal threshold (reference range: 160-450 mg/dL). Fibrinogen activity levels were measured by the Clauss method and the antigen levels by the turbidimetric latex immunoassay (HYPHEN BioMed, LIAPHEN). For molecular analysis, samples were sent to the Angelo Bianchi Bonomi Hemophilia and Thrombosis Center (Fondazione IRCCS Ca’ Granda Ospedale Maggiore Policlinico) or the Department of Genetic Medicine and Development in Geneva, and direct sequencing of amplified fragments was performed by Sanger sequencing. Genetic variants were classified according to the guidelines established by the American College of Medical Genetics and Genomics [19]. Data were collected from centers across 8 countries, primarily in Europe (Table). The severity of bleeding was evaluated according to the criteria of the European Network of Rare Bleeding Disorders [20]. HMB was defined as blood loss > 80 mL per cycle, with variation possible depending on each center’s protocols [21]. PPH was defined as an estimated blood loss > 500 mL after vaginal delivery or > 1000 mL after cesarean delivery [22]. The project was approved by the ethical review board of the Fondazione IRCCS Ca’ Granda Ospedale Maggiore Policlinico, Milan, Italy and written informed consent was obtained from all enrolled patients or their parents.

**Table:** Obstetric complications and characteristics of women who experienced pregnancy.

Case	Disorder	Age at inclusion	Pregnancy (*n*)	Miscarriage (*n*)	Miscarriage trimester	Live births	Fibrinogen activity (mg/dL)^a^^,^^b^	cDNA	Protein	Gene	transcript	Genotype^c^	Exon/ intron	ACMG^d^ classification	Country
1	Dysfibrinogenemia	32	1	1	NA^e^	0	152	c.1410dupT	p.Gly471TrpfsTer3	*FGA*	Frameshift	Hetero	5	Likely pathogenic	Italy
2	Dysfibrinogenemia	49	2	1	NA	1	35	c.114G>C	p.Arg38Ser	*FGA*	Missense	Hetero	2	Uncertain_significance	Italy
3	Dysfibrinogenemia	52	1	0	NA	1	66	c.901C>T	p.Arg301Cys	*FGG*	Missense	Hetero	9	Pathogenic	Italy
4	Dysfibrinogenemia	41	1	0	NA	1	71	c.902G>A	p.Arg301His	*FGG*	Missense	Hetero	8	Pathogenic	Italy
5	Dysfibrinogenemia	67	2	0	NA	2	124	c.1410dupT	p.Gly471TrpfsTer3	*FGA*	Frameshift	Hetero	5	Likely pathogenic	Italy
6	Dysfibrinogenemia	25	3	1	After the first trimester	2	106	NA	NA	NA	NA	NA	NA	NA	US
7	Dysfibrinogenemia	25	1	1	NA	0	61	NA	NA	NA	NA	NA	NA	NA	US
8	Dysfibrinogenemia	73	1	0	NA	1	74	c.902G>A	p.Arg301His	*FGG*	Missense	Hetero	8	Pathogenic	Italy
9	Dysfibrinogenemia	28	6	2	Before the first trimester	4	91	NA	NA	NA	NA	NA	NA	NA	US
10	Dysfibrinogenemia	49	3	0	NA	3	52	c.112A>G	p.Arg38Gly	*FGA*	Missense	Hetero	2	Pathogenic	Italy
11	Dysfibrinogenemia	44	1	0	NA	1	77	c.112A>G	p.Arg38Gly	*FGA*	Missense	Hetero	2	Pathogenic	Italy
12	Dysfibrinogenemia	39	2	0	NA	2	139	NA	NA	NA	NA	NA	NA	NA	Italy
13	Dysfibrinogenemia	50	3	1	Before the first trimester	2	42	c.1151C>G	p.Ser384Cys	*FGG*	Missense	Hetero	9	Uncertain significance	Italy
14	Dysfibrinogenemia	61	1	0	NA	1	48	NA	NA	NA	NA	NA	NA	NA	UK
15	Dysfibrinogenemia	76	4	1	After the first trimester	3	45	c.901C>T	p.Arg301Cys	*FGG*	Missense	Hetero	8	Pathogenic	UK
16	Dysfibrinogenemia	49	4	2	Before the first trimester	2	48	c.901C>T	p.Arg301Cys	*FGG*	Missense	Hetero	8	Pathogenic	Italy
17	Dysfibrinogenemia	28	1	0	NA	1	37	NA	NA	NA	NA	NA	NA	NA	UK
18	Dysfibrinogenemia	40	5	3	Before the first trimester	2	60	NA	NA	NA	NA	NA	NA	NA	Switzerland
19	Dysfibrinogenemia	39	2	1	NA	1	50	NA	NA	NA	NA	NA	NA	NA	Switzerland
20	Dysfibrinogenemia	36	2	0	NA	2	86	c.902G>A	p.Arg301His	*FGG*	Missense	Hetero	8	Pathogenic	Italy
21	Dysfibrinogenemia	46	4	2	NA	2	51	c.901C>T	p.Arg301Cys	*FGG*	Missense	Hetero	8	Pathogenic	Italy
22	Dysfibrinogenemia	51	1	1	NA	0	72	c.112A>G	p.Arg38Gly	*FGA*	Missense	Hetero	2	Pathogenic	Italy
23	Dysfibrinogenemia	29	3	3	Before the first trimester	0	60	NA	NA	NA	NA	NA	NA	NA	Serbia
24	Hypofibrinogenemia	30	2	0	NA	2	33	c.1001G>A	p.Trp334Ter	*FGA*	Nonsense	Homo	4	Likely pathogenic	The Netherlands
25	Hypofibrinogenemia	38	1	0	NA	1	90	c.490G>A	p.Asp164Asn	*FGB*	Nonsense	Hetero	3	Likely pathogenic	The Netherlands
26	Hypofibrinogenemia	26	2	0	NA	2	73	c.180+1G>C	-	*FGA*	Splice site	Hetero	Int2	Uncertain_significance	The Netherlands
27	Hypofibrinogenemia	61	1	0	NA	1	107	c.1024G>A	p.Asp342Asn	*FGG*	Missense	Hetero	8	Likely pathogenic	Italy
28	Hypofibrinogenemia	37	1	0	NA	1	90	c.998A>G	p.His333Arg	*FGG*	Missense	Hetero	8	Likely pathogenic	Greece
29	Hypofibrinogenemia	22	4	3	Before the first trimester	1	75	NA	NA	NA	NA	NA	NA	NA	Iran
30	Hypofibrinogenemia	38	2	1	Before the first trimester	1	130	c.709T>C	p.Tyr237His	*FGG*	Missense	Hetero	7	Likely pathogenic	Switzerland
31	Hypofibrinogenemia	37	1	0	NA	1	51	NA	NA	NA	NA	NA	NA	NA	Italy
32	Hypofibrinogenemia	36	2	1	Before the first trimester	1	110	NA	NA	NA	NA	NA	NA	NA	Italy

ACMG, American College of Medical Genetics and Genomics; cDNA, complementary DNA; Int, intron.

a Fibrinogen activity reference range: 160-450 mg/dL. Fibrinogen activity levels were recorded at the time of inclusion.

b Factor activity levels were measured using the Clauss method.

c Homo, homozygous; Hetero, heterozygous.

d The ACMG classification is a standardized framework for interpreting genetic variants, particularly with regard to their potential pathogenicity.

e NA, not available.

### 2.1 Statistical analysis

The chi-squared test was used to compare the rate of menorrhagia and obstetric complications across different groups of CFDs. The Mann–Whitney *U*-test was applied to compare baseline factor activity levels between live birth and complicated pregnancies. Due to the small sample size, we performed Fisher’s exact test to assess the potential association between patients carrying hotspot variants and those without. The analyses were conducted using RStudio (2022.7.1 “Foundation” release) as the integrated development environment for all R-based computations and Excel 2016 (Microsoft).

## 3 Results

### 3.1 Population

**Figure 1:**
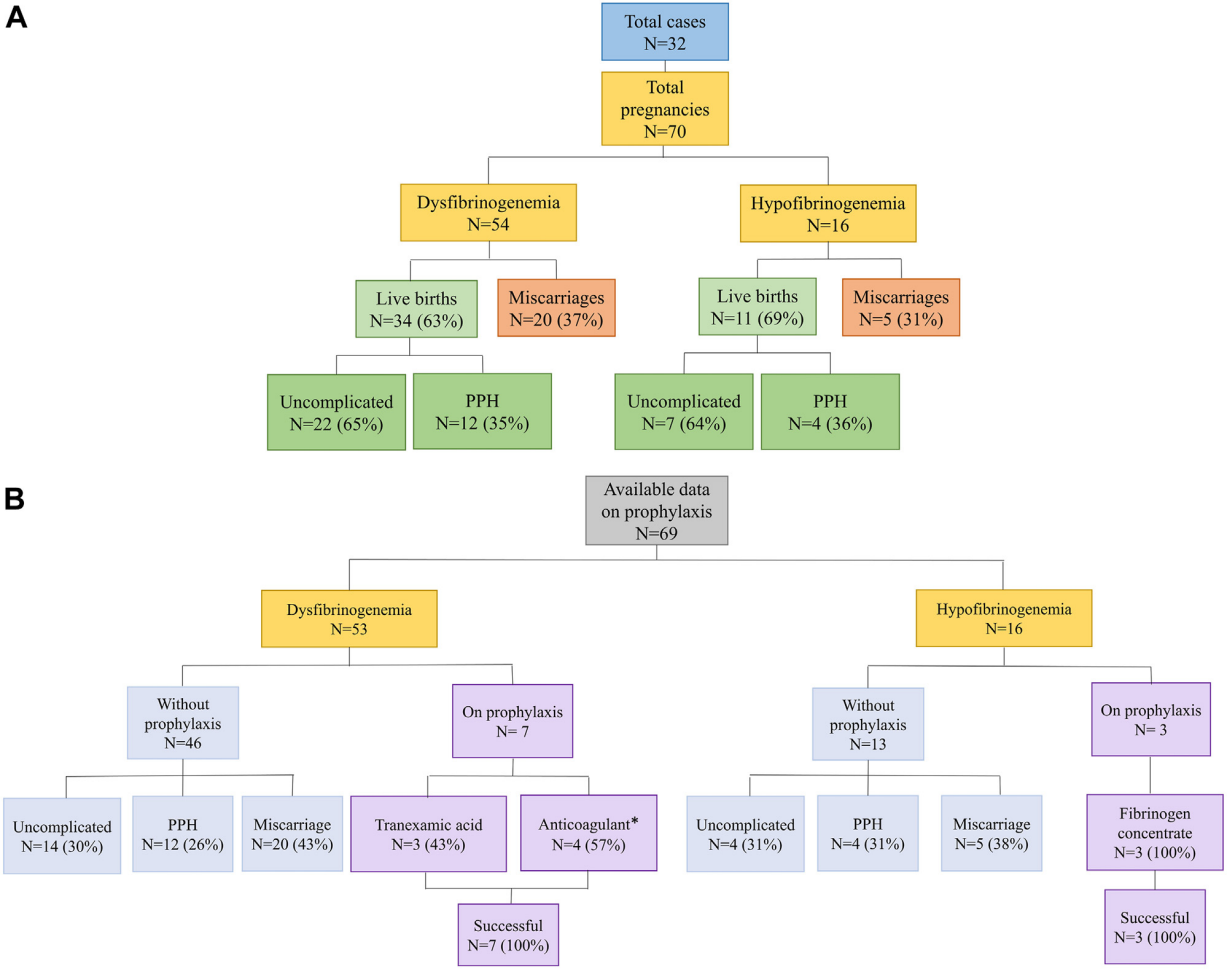
Flowchart of study population selection, including total pregnancies and prophylaxis status. (A) Flowchart of pregnancy outcomes in patients with dysfibrinogenemia and hypofibrinogenemia. The outcomes are further divided into live births, PPH and miscarriages. (B) Comparison of pregnancy outcomes between cases managed with prophylaxis and those without prophylaxis. ^∗^Four patients received thromboprophylaxis (unfractionated and low molecular weight heparin) because of previous miscarriages. PPH, postpartum hemorrhage.

In this study, we included 59 adult females (≥12 years old) consisting of 8 with afibrinogenemia, 15 with hypofibrinogenemia, and 36 with dysfibrinogenemia. Among them, 33 women (56%) had a total of 71 pregnancies, including 1 pregnancy in afibrinogenemia, 16 pregnancies in hypofibrinogenemia, and 54 in dysfibrinogenemia. The only recorded pregnancy in an afibrinogenemic case resulted in miscarriage and was not managed with prophylactic therapy. Given the relatively low median age of the patients in this study (21 years, range: 12-47 years), the number of pregnancies observed was limited. This small number of pregnancies led to the exclusion of pregnancy outcomes in afibrinogenemia from further analysis. Therefore, we chose to describe obstetric complications between hypofibrinogenemia and dysfibrinogenemia (women, *n* = 32, pregnancies, *n* = 70) (Figure 1A).

Fibrinogen activity levels were assayed at the time of inclusion with fibrinogen activity levels < 10 mg/dL in afibrinogenemia, median factor activity levels of 90 mg/dL (range: 33-130 mg/dL) in hypofibrinogenemia and 60 mg/dL (range: 30-150 mg/dL) in dysfibrinogenemia. The median fibrinogen antigen levels in hypofibrinogenemia and dysfibrinogenemia were 118 mg/dL (range: 27-155 mg/dL) and 264 mg/dL (range: 184-438 mg/dL), respectively. Considering women with ≥1 pregnancy, genetic data were available for 15 dysfibrinogenemic and 5 hypofibrinogenemic cases.

### 3.2 Heavy menstrual bleeding

Of the 59 analyzed women, 21 (36%) experienced HMB. Among the 8 afibrinogenemic cases, 4 (50%) were receiving prophylaxis. Of those who did not receive prophylaxis, 3 (75%) experienced HMB. However, there is no significant difference between the proportions of HMB in hypofibrinogenemic (4/15, 27%) and dysfibrinogenemic (13/36, 36%) cases (*P* = .78).

### 3.3 Obstetric complications

Considering all cases with ≥1 pregnancy, a total of 23 dysfibrinogenemic cases with 54 pregnancies and 9 hypofibrinogenemic cases with 16 pregnancies were analyzed (Figure 1A). Among the 70 pregnancies, 45 (64%) resulted in live births, of which 16 (36%) were complicated by PPH, and none were receiving prophylaxis. Meanwhile, 25 (36%) pregnancies ended in miscarriage with recurrent pregnancy loss observed in 6 (38%) cases, accounting for 60% of all miscarriages (Table). Of note, among the available data on the timing of miscarriages, 8 of 10 patients (80%) experienced ≥1 miscarriage during the first trimester of pregnancy.

Of the 54 pregnancies of individuals with dysfibrinogenemia, 34 (63%) resulted in live births, with 22 (65%) being uncomplicated and 12 (35%) complicated by PPH. Additionally, 20 (37%) pregnancies ended in miscarriage.

Similarly, among the 16 pregnancies in individuals with hypofibrinogenemia, 11 (69%) resulted in live birth, with 7 (64%) being uncomplicated and 4 (36%) complicated by PPH. Moreover, 5 pregnancies (31%) resulted in miscarriage. Similarly, among the 16 pregnancies in individuals with hypofibrinogenemia, 11 (69%) resulted in live birth, with 7 (64%) being uncomplicated and 4 (36%) complicated by PPH. Moreover, 5 pregnancies (31%) resulted in miscarriage. When considering the severity of hypofibrinogenemia based on fibrinogen activity levels—severe (< 50 mg/dL), moderate (50-100 mg/dL), and mild (> 100 mg/dL up to the lower limit of the laboratory reference range)—only 1 severe case was reported, in which both pregnancies resulted in successful deliveries, possibly due to prophylactic fibrinogen replacement therapy. In moderate and mild cases, live birth rates were 67% (*n* = 6) and 60% (*n* = 3), respectively, while miscarriage occurred in 33% (*n* = 3) and 40% (*n* = 2) of cases. We found no significant difference in the rates of PPH and miscarriage between cases with dysfibrinogenemia and hypofibrinogenemia (*P* = .66).

Among the 23 dysfibrinogenemic cases, 5 of them with a total of 10 pregnancies were asymptomatic before pregnancy. Of these, 5 pregnancies (50%) resulted in uncomplicated live births (cases, *n* = 3), while the remaining 5 (50%) resulted in miscarriage (cases, *n* = 3). Notably, 1 individual experienced both successful live births and complicated pregnancies.

### 3.4 Genetic variants and pregnancy complications

Of the 32 women who experienced pregnancies, genetic data were available for 22 (69%), covering a total of 41 pregnancies: 32 in dysfibrinogenemia and 9 in hypofibrinogenemia. In dysfibrinogenemic cases, 9 of 16 (56%) carried hotspot variants (p.Arg301Cys/His) in *FGG* exon 8 (Table). Among their 19 pregnancies, 14 (74%) resulted in live births; 6 (43%) of these pregnancies were uncomplicated while 8 (57%) were complicated with PPH. Notably, both hotspot variants are classified as pathogenic according to American College of Medical Genetics and Genomics guidelines. The remaining 5 (29%) pregnancies resulted in miscarriage. Nevertheless, there was no statistically significant difference in the rates of miscarriage and PPH between individuals with and without hotspot variants (*P* = .94).

### 3.5 Fibrinogen replacement prophylaxis and thromboprophylaxis

Among 31 cases with available prophylaxis data, 10 pregnancies received prophylaxis, all resulting in successful outcomes (Figure 1B). Among 7 treated pregnancies in dysfibrinogenemic cases, 3 received tranexamic acid, while the remaining 4 were managed solely with antithrombotic prophylaxis (unfractionated heparin and low molecular weight heparin) after previous recurrent miscarriages. All pregnancies proceeded without PPH. All 3 pregnancies in women with hypofibrinogenemia were treated with fibrinogen concentrate, likely due to a history of bleeding diathesis, including hematoma, intermenstrual vaginal bleeding, and bleeding after tooth extraction.

**Figure 2:**
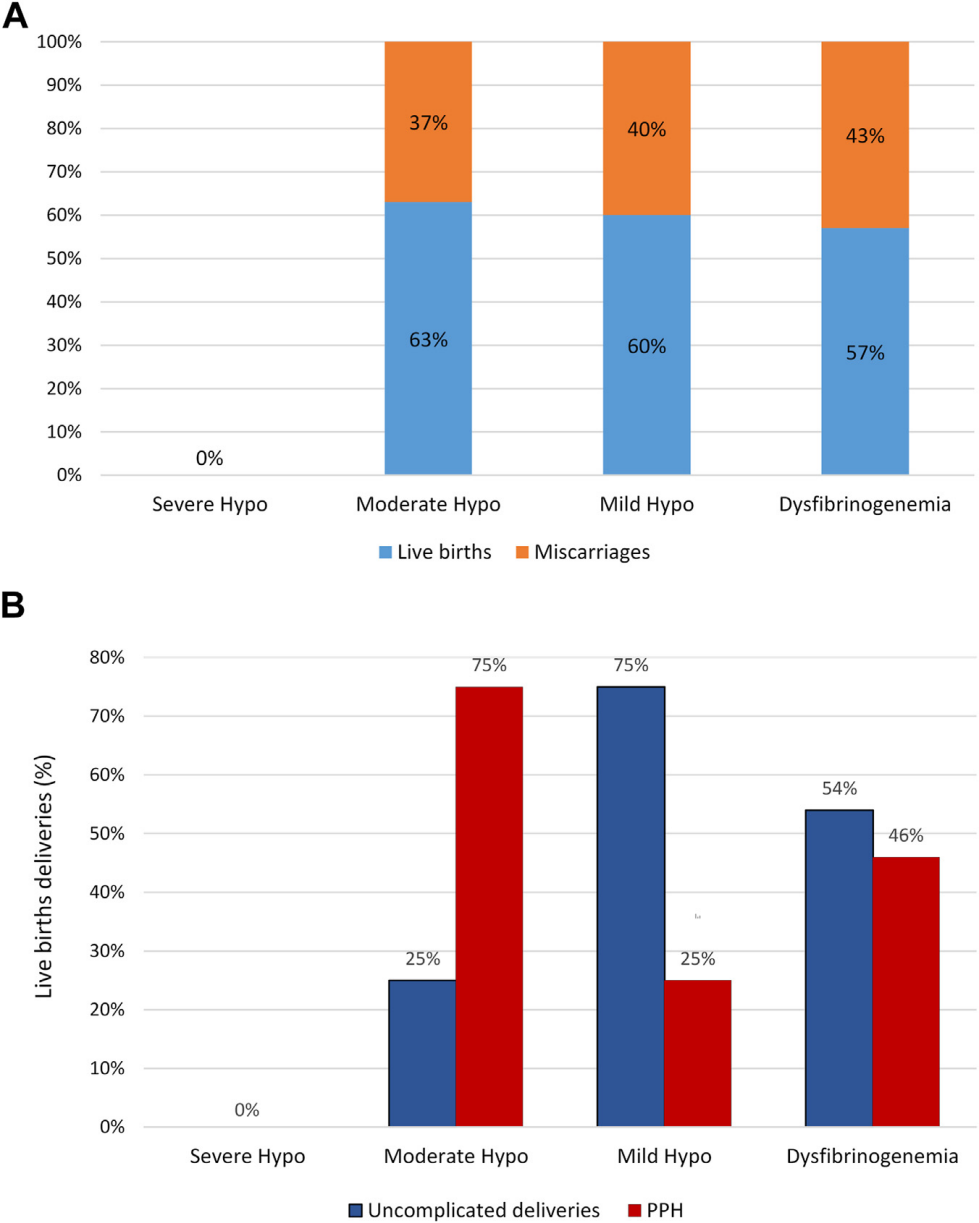
Comparison of pregnancy outcomes between dysfibrinogenemic and hypofibrinogenemic cases without prophylaxis. (A) The chart shows the percentage of live births and miscarriages for each condition. The severity of hypofibrinogenemia is based on the fibrinogen activity level (severe: < 50 mg/dL; moderate: 50-100 mg/dL; mild: > 100 mg/dL to the lower limit of laboratory reference range). (B) Considering live births in patients who were not receiving prophylaxis, the percentages of uncomplicated deliveries and those complicated by PPH were compared between dysfibrinogenemia and hypofibrinogenemia. There was 1 case of severe hypofibrinogenemia with 2 successful deliveries, both of which occurred under prophylaxis. hypo, hypofibrinogenemia; PPH, postpartum hemorrhage.

In contrast, of the 59 pregnancies (46 in dysfibrinogenemia and 13 in hypofibrinogenemia) not managed with prophylaxis, 34 (58%) resulted in live births, of which 18 (53%) were without complications, 16 (47%) were complicated by PPH, and 25 (42%) of all pregnancies ended in miscarriage, with 75% occurring in the first trimester (Figure 2A, B).

Among 53 pregnancies in patients with dysfibrinogenemia, 46 were not managed with prophylaxis. Of 26 live birth deliveries, 14 (54%) had a successful outcome, 12 (46%) resulted in PPH, and 20 (43%) pregnancies ended in miscarriage (Figure 1B). Similarly, among 16 pregnancies in hypofibrinogenemic patients, 13 were not managed with prophylaxis. Among 8 live births, 4 (50%) had a successful outcome, 4 (50%) resulted in PPH, and 5 (38%) pregnancies ended in miscarriage (Figure 2A, B).

## 4 Discussion

Women with inherited bleeding disorders are at an increased risk of HMB and pregnancy-related bleeding; however, data on obstetric complications in cases of CFDs remain limited. This study, as part of the PRO-RBDD cohort, investigated HMB and obstetric complications in individuals with CFDs, with a particular focus on dysfibrinogenemia and hypofibrinogenemia. As afibrinogenemic cases are generally more severe and typically diagnosed at an early age, the cases included in this study also had a relatively low median age of 21 years, which may have contributed to the limited number of pregnancies observed.

The rate of HMB did not differ significantly between dysfibrinogenemic and hypofibrinogenemic cases, with reported rates of 36% and 27%, respectively. Similar to our previous study, HMB was identified as the most prevalent symptom in CFD cases, with a prevalence of 33% [23]. However, the rate of HMB in afibrinogenemic patients who did not receive prophylaxis was 75%. Similarly, in both the RBiN (Rare Bleeding Disorders in the Netherlands) and QualyAfib (Quality of Life in Patients With Congenital Afibrinogenemia) studies, HMB was reported in over 50% and 73.7% of the CFD population, respectively [24,25]. This suggests that the occurrence of HMB in individuals with afibrinogenemia, particularly those are not on prophylactic treatment, may be a common and significant clinical feature across different patient cohorts.

The percentages of live birth and PPH in our study were 64% and 23%, respectively, which are almost comparable to the findings of a previous study by Hugon-Rodin et al. [11] (74% and 20%) and notably higher than the PPH rate in the general population (1%-10%) [26]. Consistent with findings from this study, the prevalence of live births in cases of dysfibrinogenemia (63%) and hypofibrinogenemia (69%) was comparable (76.2% vs 71.1%) [11]. However, live births were slightly more frequent in cases of hypofibrinogenemia.

Among the total deliveries in dysfibrinogenemia, 22% resulted in PPH, with none of these cases receiving treatment. This rate is similar to that observed in 2 previous studies, which reported PPH rates of 21% and 20.7% [5,11]. In the Fibrinogest study, the rate of PPH in women with hypofibrinogenemia was reported at 18.2%, which was lower than the rate observed in our study [11]. However, considering live births, the comparable rates of PPH for dysfibrinogenemia (35%) and hypofibrinogenemia (36%) highlight that both conditions pose substantial risks during pregnancy, emphasizing the need for vigilant monitoring and timely intervention. Notably, bleeding during pregnancy occurred exclusively in dysfibrinogenemic cases, albeit with a low frequency (*n* = 2, 4%), and none of these cases were on thromboprophylaxis.

In this study, the rate of miscarriage (36%) was higher than those observed in both the European and North American general populations (10%-20%) and the Fibrinogest study (13%) [11,27,28]. However, it was lower than that reported by Haverkate et al. [15] (47%), likely due to the inclusion of patients with thrombotic-related fibrinogen variants in their cohort. In a previous study by Casini et al. [5], the miscarriage rate in dysfibrinogenemia was 19.8% based on 111 pregnancies. Our study observed a higher rate of 37%, possibly due to the inclusion of more severe cases with greater obstetric complications. According to a few documented case reports, hypofibrinogenemia has been associated with recurrent miscarriages, although at a lower rate than other CFDs [29]. Nevertheless, the higher rate observed in this study (31%) underscores the critical need for careful management of pregnancies in affected women.

Interestingly, pregnancies in asymptomatic cases with dysfibrinogenemia were associated with miscarriage (50%). This suggests that pregnancy can lead to complications even in asymptomatic individuals with dysfibrinogenemia. Therefore, asymptomatic cases with dysfibrinogenemia should also be considered for routine follow-up and may benefit from prophylactic fibrinogen replacement therapy during pregnancy.

Baseline factor activity levels showed no significant difference between women with CFD with live births and those with miscarriages, nor between those who experienced PPH and those who did not (*P =* .98 and *P* **=** .69, respectively). Due to the limited sample size, these findings were not included in the results. Therefore, further studies focused specifically on a large number of patients with hypofibrinogenemia could provide valuable insights into the role of factor activity in predisposing individuals to obstetric complications.

The hotspot variants p.Arg301His/Cys in *FGG* were identified in 56% of dysfibrinogenemic cases. Although these cases experienced obstetric complications, with 57% experiencing PPH and 29% of pregnancies resulting in miscarriage, no significant difference was found between cases with and without hotspot variants (*P =* .94). Similarly, in a cohort of 101 dysfibrinogenemia cases, hotspot variants were not found to be associated with pregnancy complications [5].

The findings from this study showed the critical importance of prophylactic measures in improving pregnancy outcomes for women with CFDs. Since only 39% of those who were not on prophylaxis achieved successful outcomes, this demonstrates the considerable risks associated with inadequate management of CFDs during pregnancy. The high rates of miscarriage (42%) and PPH (27%) in the nonprophylaxis group further emphasize the vulnerability of these patients. Interestingly, the absence of a significant difference in outcomes between hypofibrinogenemic and dysfibrinogenemic cases without prophylaxis suggests that both conditions present comparable risks when left untreated. Therefore, although the rate of live births in individuals with CFDs was high (64%), these pregnancies were still associated with complications. This highlights the potential benefit of prophylactic measures in preventing severe outcomes, taking into account both the family and the patient’s history of bleeding or thrombotic events.

The management of dysfibrinogenemia during pregnancy poses unique challenges due to the risk of both bleeding and thrombosis [30]. Our dysfibrinogenemic patient, who had successful deliveries with thromboprophylaxis, suggests that careful selection and consideration of anticoagulant therapy can contribute to positive pregnancy outcomes in women with dysfibrinogenemia, particularly those with a history of recurrent miscarriages. However, due to the rarity and variability of this condition, a tailored approach should be based on a thorough multidisciplinary evaluation, with family history playing a key role in guiding treatment decisions.

This study had several limitations, including incomplete data on prophylaxis, the timing of miscarriages, lack of information on the type of delivery, and the absence of genetic results for some cases. Additionally, the low sample size limited our ability to assess the association between fibrinogen concentration and the risk of obstetric complications. This also hindered our ability to determine threshold levels required to prevent bleeding and miscarriages. Nevertheless, maintaining fibrinogen activity levels > 100 mg/dL during pregnancy and > 150 mg/dL during labor is recommended [13]. In addition, there may be a potential bias due to the inclusion of a higher proportion of symptomatic women, although 22% of the cases were diagnosed incidentally, either before surgery or through familial analysis.

## 5 Conclusion

This study examined HMB and obstetric complications in women with CFDs, analyzing data from 70 pregnancies as part of the PRO-RBDD study. The HMB occurrence did not differ between patients with hypofibrinogenemia and those with dysfibrinogenemia. The rate of miscarriage and PPH in women with CFDs was substantially higher than that observed in the European and North American populations. Although live birth rates are high in women with hypofibrinogenemia and dysfibrinogenemia, pregnancies can still be complicated by obstetric challenges, even in asymptomatic cases. In this study, no significant difference in obstetric complications was found between afibrinogenemia and hypofibrinogenemia. Early CFD diagnosis before pregnancy, combined with the appropriate prescription of procoagulant and/or anticoagulant prophylaxis, may serve as an effective preventive strategy.
